# Simple divalent metal salts as robust and efficient initiators for the ring-opening polymerisation of *rac*-lactide†

**DOI:** 10.1039/d4ra07747d

**Published:** 2024-11-29

**Authors:** Phoebe A. Lowy, Jennifer A. Garden

**Affiliations:** a EaStCHEM School of Chemistry, University of Edinburgh Edinburgh EH9 3FJ UK j.garden@ed.ac.uk

## Abstract

Simple divalent metal benzoxides are reported as robust and efficient catalysts for lactide (LA) polymerisation. Following a “pre-stir” step to aid solubility, the best performing catalyst, Zn(OBn)_2_, gave quantitative monomer conversion in just 30 seconds and performed well under industrially relevant settings with high monomer loadings, bulk polymerisation conditions and non-anhydrous conditions using technical grade LA.

## Abstract 2

Simple salts such as Zn(OBn)_2_ are robust and efficient initiators for lactide polymerisation, delivering poly(lactic acid) under industrially relevant conditions.

## Graphical abstract

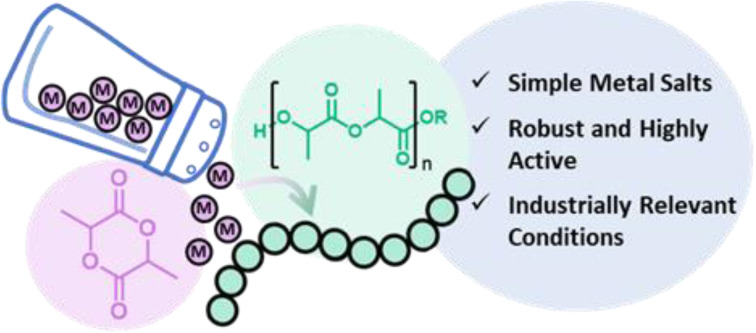

Given the high societal reliance of plastics, there is a major push to develop sustainable alternatives to petrochemical-derived polymers.^1^ One of the most promising replacements is poly(lactic acid) (PLA), a bio-derived polyester that is biodegradable, recyclable, and has diverse applications in packaging, drinking cups, bottles, fibres and medical sutures/stents.^2,3^ PLA is typically produced *via* the ring-opening polymerisation (ROP) of lactide (LA) using tin(ii) octanoate (Sn(Oct)_2_, Fig. 1A),^6–8^ which is the standard industry catalyst due to its high activity.^4,9^ However, the drawbacks include toxicity concerns that limit certain applications within the biomedical field, and the need for high monomer purity because water and other impurities can reduce the polymerisation control.

**Fig. 1:**
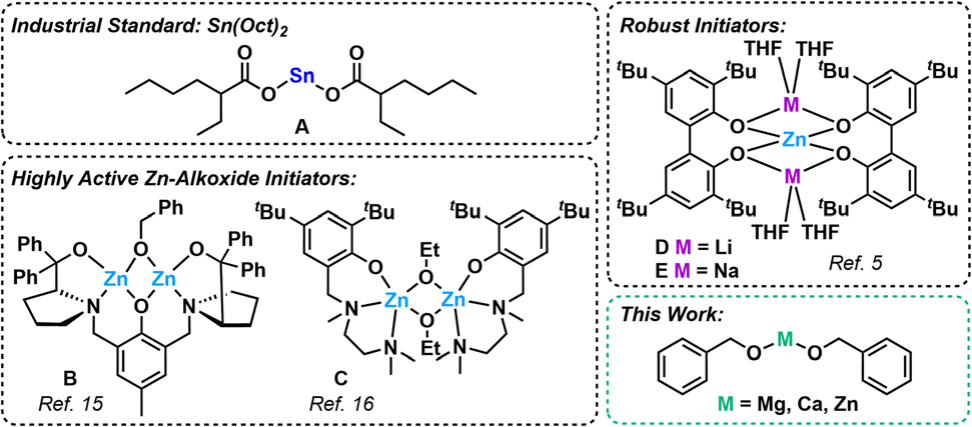
Examples of the industrial standard Sn(Oct)_2_, highly active and/or robust Zn-alkoxide initiators, and the simple salts explored for LA ROP in this work.^4,5,14,15^

While many alternatives to Sn(Oct)_2_ have been reported, most rely on carefully designed ligands to boost the activity, by improving the solubility, fine-tuning the electronics or changing the metal coordination environment.^10^ Experimental and theoretical studies have shown that ligand-supported monometallic catalysts for cyclic ester ROP generally proceed *via* a coordination–insertion mechanism (Scheme 1).^7,11^ Polymerisation occurs *via* monomer coordination to the Lewis acidic metal centre with nucleophilic attack, ring-opening and insertion of the cyclic ester into a metal-O_alkoxide_ bond. Some of the most efficient metal-based initiators reported for cyclic ester ROP are complexes based on magnesium, calcium and especially zinc (see Fig. 1B, C and S13† for some representative examples).^4,12–16^ Zinc is particularly attractive because it is inexpensive, colourless, non-toxic, and relatively robust towards air and moisture compared to Mg and Ca.^12,13,17^ However, such divalent metal complexes typically require multi-step syntheses and are air- and moisture-sensitive, thus need to be handled under extremely anhydrous conditions. This limits their use in large scale, industrially relevant settings, and so the development of robust, air- and moisture-tolerant analogues is highly appealing. While there are relatively few examples of robust LA ROP catalysts based on low toxicity metals,^18^ Wu and co-workers reported two air-stable heterometallic Li/Zn and Na/Zn catalysts (Fig. 1D and E).^5^ Notably, E was active for l-LA ROP when the reactions were performed in air using an unpurified monomer source, giving PLA with controllable molecular weight and high LA conversions of 94%, albeit after a relatively long reaction time (48 h, *Đ* = 1.4, 90 °C, toluene, [cat] : [l-LA] 1 : 175).

**Scheme 1:**
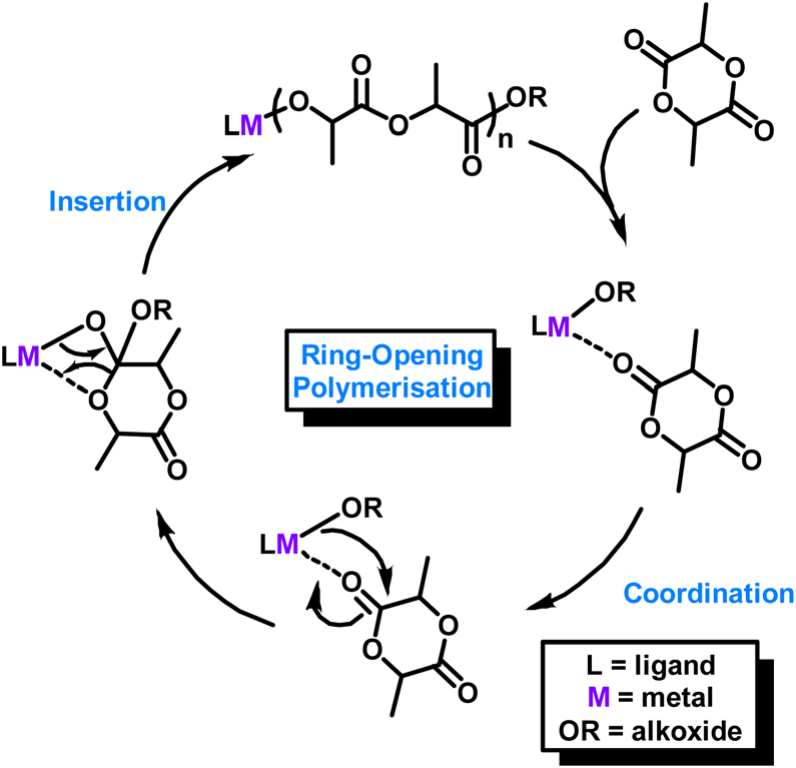
Proposed coordination insertion mechanism for LA ROP using a monometallic catalyst.

In terms of simple, non-ligated initiators for cyclic ester ROP, metal-alkyl reagents such as BuLi and Bu_2_Mg have been reported. While these are efficient initiators, and can even deliver partial heterotacticity control in *rac*-LA ROP, they are also highly pyrophoric. Alternatively, simple metal-alkoxide salts, including those based on Li,^19^ Mg,^20^ Ca,^21^ Al,^22,23^ Y^24^ and La^25^ have been exploited as efficient initiators for LA ROP, including those added as activity boosters to harness heterometallic cooperativity in mixed-metal catalyst systems.^26^ Beyond lactide,^27^ the use of simple zinc salts has been demonstrated in the ROP of other cyclic esters, including the use of Zn lactate in the ROP of 1,4-dioxan-2-one,^28^ and Zn(OBn)_2_ for the ROP of gem-disubstituted valerolactones.^29^ Herein, we report the use of three simple metal salts based on low toxicity metals, Mg(OBn)_2_, Ca(OBn)_2_, and Zn(OBn)_2_, as highly active initiators in LA ROP upon incorporation of a “pre-stir” into the reaction set-up, and probed the robustness of these systems towards industrially relevant conditions.

Metal-alkoxides Mg(OBn)_2_, Ca(OBn)_2_, and Zn(OBn)_2_ were generated from the addition of 2 equiv. of benzyl alcohol (BnOH) to Mg(HMDS)_2_, Ca(HMDS)_2_(THF)_2_ or Et_2_Zn in THF solvent at room temperature.^26^ BnOH was selected because it is inexpensive, commercially available, and ligated metal-benzoxides have shown good activities in cyclic ester ROP. The complexes were characterised by NMR spectroscopy and MALDI-ToF mass spectrometry but unfortunately, attempts to crystallise these three complexes from a range of solvents including THF, toluene and chloroform were unsuccessful. ^1^H NMR characterisation revealed the presence of 1 equiv. of residual HMDSH in isolated Mg(OBn)_2_ (which was factored into the MW), and trace HMDSH in Ca(OBn)_2_ (∼0.1 equiv.). As removal of residual HMDSH has previously been reported to decompose group 2 complexes, Mg(OBn)_2_ and Ca(OBn)_2_ were employed directly as catalysts.^17^ While Mg(OBn)_2_, Ca(OBn)_2_, and Zn(OBn)_2_ have been previously reported for *rac*-lactide polymerisation (r.t., THF solvent), Zn(OBn)_2_ was completely inactive.^26^ Here, all three M(OBn)_2_ salts were tested for *rac*-LA ROP at 70 °C in toluene with 1 M LA concentration; conditions that have been successful for other ROP catalysts.^17^ Under these conditions, all three M(OBn)_2_ salts were active in *rac*-LA ROP, with reasonable polymerisation control (Table 1, entries 1–4). Zn(OBn)_2_ and Mg(OBn)_2_ converted 50 equiv. and 140 equiv. of *rac*-LA in 40 min, respectively (entries 1 and 2), whilst Ca(OBn)_2_ was the most active and converted 90 equiv. of LA in just 2 min (entry 3).

**Table 1:** Optimisation of polymerisation conditions using M(OBn)_2_ salts for *rac*-LA ROP^a^

Entry	Cat.	LA equiv.	Pre-stir (min)	Time (min)	Conv.^b^ (%)	*k*_obs_ (min^−1^)	*M*_n calc_ c (kg mol^−1^)	*M*_n obs_ d (kg mol^−1^)	*M*_n obs_/*M*_n calc_ (2 chains)	*Đ*
1	Zn(OBn)_2_	100	—	40	50	0.025	3.6	4.8	1.35	1.18
2	Mg(OBn)_2_	167	—	40	84	0.07	10.1	9.0	0.88	1.40
3	Ca(OBn)_2_	100	—	2	90	—	6.5	6.8	1.04	1.62
4	Ca(OBn)_2_	100	—	5	97	—	7.0	4.3	0.62	2.65
5	Zn(OBn)_2_	100	30	0.5	96	6.0	6.9	7.0	1.00	1.47
6	Mg(OBn)_2_	167	30	5	91	0.49	10.9	8.3	0.68	1.99
7	Mg(OBn)_2_	100	30	5	93	0.49	6.7	7.1	1.05	1.52
8	Ca(OBn)_2_	100	30	2	87	0.81	6.3	5.4	0.90	1.53
9^e^	HMDSH	100	—	1440	0	—	—	—	—	—

a [*rac*-LA] = 1 M in toluene, 70 °C. For Mg(OBn)_2_, the mass of residual HMDSH (1.0 equiv.) was factored into the catalyst mass.

b Conversion calculated using ^1^H NMR spectroscopy.

c *M*_n calc_ of polymers calculated from *M*_n calc_ = *M*_0_ × ([*M*]/[*I*]) × conversion assuming 2 chains per catalyst.

d *M*_n obs_ and *Đ* determined by SEC using polystyrene standards in THF. Values corrected by a correction factor of 0.58.^21^

e [HMDSH] : [BnOH] : [*rac*-LA] = 1 : 1 : 100.

Performing kinetic studies with the slower Zn(OBn)_2_ and Mg(OBn)_2_ catalysts revealed an induction period of approximately 10 min in both cases (Fig. S4†). The analysis of induction periods is important because catalysts can undergo changes during this time, such as a change in the aggregation state, solubilisation of the catalyst species, structural rearrangement of a ligand to facilitate metal coordination,^15^ or alcoholysis of a metal-alkyl precursor to generate an active metal-alkoxide initiator. Introducing a “pre-stir” step, in this case to aid solubulity of both the catalyst species and monomer, can overcome the induction period, giving a better representation of the true propagation rate.^30^ Therefore, two separate solutions of M(OBn)_2_ in toluene and *rac*-LA in toluene were stirred for 30 min at 70 °C, using a “pre-stir” step to ensure *rac*-LA dissolution and aid catalyst solubility before the catalyst was added to the LA solution (Table 1, entries 5–8). All three salts gave first order kinetics with respect to monomer concentration (Fig. S5†). For Zn(OBn)_2_, there was a remarkable increase in rate after introducing this 30 minutes “pre-stir”; the *k*_obs_ value increased by a factor of 240 (Table 1, entries 5 *vs.* 1). The activity of Mg(OBn)_2_ also improved, albeit to a lesser extent, with the observed rate constant increasing by a factor of 7 (entries 6 *vs.* 2). In contrast, Ca(OBn)_2_ gave similarly high *rac*-LA conversions with or without a pre-stir (90% *vs.* 87% in 2 min, respectively, entries 3 and 8). Identical polymerisation rates were observed whether a 1 : 167 or 1 : 100 loading of Mg(OBn)_2_ : *rac*-LA was used (both *k*_obs_ = 0.49 min^−1^, Table 1 entries 6 and 7), where the 1 equiv. of residual HMDSH present was factored in to the catalyst MW in both cases. Note that if the mass of HMDSH was not included in the catalyst MW, the 1 : 167 ratio becomes a 1 : 100 ratio. The identical *k*_obs_ values therefore imply that, under these conditions, factoring residual HMDSH into the calculations does not significantly impact the catalyst activity. No HMDS-capped PLA was detected by MALDI-ToF analysis, and a control reaction confirmed that HMDSH does not initiate *rac*-LA ROP in the presence of BnOH (entry 9 and Fig. S8†). Taken together, the data shows that HMDSH does not initiate *rac*-LA ROP in these systems, which is consistent with literature reports for other divalent metal catalysts.^17,26^

For all three M(OBn)_2_ salts, *M*_n obs_ generally increased linearly with conversion (Fig. S7†), although Zn(OBn)_2_ gave some lower-than-expected *M*_n obs_ values in the late stages of the polymerisation. MALDI-ToF mass spectrometry was used to analyse the PLA end groups, and all three M(OBn)_2_ salts generated α-benzoxy, ω-hydroxy (major series) as well as α-hydroxy, ω-hydroxy end-capped and/or cyclic PLA as the minor series (Fig. S8†). The minor series were attributed to chain transfer or transesterification reactions, increasing in the later stages of LA polymerisation as has been reported for other catalysts.^10,31^ Taken together, the presence of OBn/H end groups and the first-order reaction in monomer supports a coordination-insertion mechanism, which has been reported for other metal salt catalysts.^32,33^ Due to the impressive activities of the M(OBn)_2_ salts in LA ROP following a “pre-stir”, these salts were further explored targeting industrially relevant conditions. The activities of the M(OBn)_2_ salts were therefore investigated at higher monomer loadings of up to 2500 equiv. of *rac*-LA in toluene at 70 °C (Table 2). Interestingly, Zn(OBn)_2_ retained excellent activities at high monomer loadings (Table 2, entries 1–7, Fig. 2a). For example, at [cat] : [*rac*-LA] loadings of 1 : 1000, Zn(OBn)_2_ converted 960 equiv. *rac*-LA in 3.5 min (*k*_obs_ = 0.94 min^−1^) achieving a high *M*_n_ value of 49.3 kg mol^−1^ (entry 5). Furthermore, Zn(OBn)_2_ tolerated even higher monomer loadings of 1 : 2500, converting 2250 equiv. of LA in 10 min (*k*_obs_ = 0.23 min^−1^), and delivered high molecular weight PLA (157.5 kg mol^−1^, entry 7). Zn(OBn)_2_ also provided excellent control with a linear increase in *M*_n obs_ and narrow dispersities (*Đ* = 1.14–1.23, entries 6–7, Fig. 2b; [*rac*-LA] = 2 M). Notably, PLA with molar mass >100 kg mol^−1^ is attractive for industrially relevant settings,^34,35^ and the dispersities achieved with Zn(OBn)_2_ are competitive with those generated using industrial catalysts (*Đ* < 2).^18,36^ While increasing the monomer loading led to a decrease in the observed rate constant, *k*_obs_, the activity of Zn(OBn)_2_ remains extremely high and is competitive with some of the most active ligand supported Zn catalysts [Fig. 2a and Table S1†].^12,13,15,16,37^ For example, under similar conditions, Zn(OBn)_2_ outperformed B (Fig. 1), with respective *k*_obs_ values of 1 × 10^−1^ s^−1^ and 3.5 × 10^−2^ s^−1^ ([cat] : [*rac*-LA] = 1 : 100, [*rac*-LA] = 1 M in toluene, 70 °C or 60 °C, respectively).^15^ Additionally, at higher monomer loadings, the activity of Zn(OBn)_2_ is approximately 20 times greater than that of the dimeric Zn complex C reported by Hillmyer, Tolman and co-workers, albeit under different reaction conditions (*k*_obs_ = 1.5 × 10^−2^ s^−1^*vs. k*_obs_ = 0.8 × 10^−3^ s^−1^, respectively; [cat] : [*rac*-LA] = 1 : 1000, [*rac*-LA] = 1 M in toluene, 70 °C *vs.* CH_2_Cl_2_, 25 °C, Fig. 1).^16^Zn(OBn)_2_ also performs well compared to other bimetallic Zn catalysts reported for higher monomer loadings (Tables 2, S1 and Fig. S13†). At a [cat] : [*rac*-LA] ratio of 1 : 600, Zn(OBn)_2_ gave 50% conversion of LA in 30 seconds at 70 °C in toluene, whereas bimetallic F (Fig. S13 and Table S1†) gave 89% conversion after 24 h in dichloromethane at 25 °C. While Zn(OBn)_2_ is highly active, it is less active than some of the best-performing Zn complexes reported to date.^12,13^ For example, under the same monomer loading of [cat] : [*rac*-LA] = 1 : 1000, the bis-Zn complex reported by Brooker, Williams and co-workers (H, Fig. S13 and Table S1†) converted 730 equiv. of LA in just 30 seconds at room temperature *vs.* 290 equiv. at 70 °C by Zn(OBn)_2_. Zn(OBn)_2_ is also less active than the guanidine hydroquinoline ligand-supported Zn complex reported by Herres-Pawlis and co-workers (I, Fig. S13 and Table S1†).^13^ However, the use of the simple Zn(OBn)_2_ salt avoids the need for extensive ligand synthesis, bringing significant advantages in terms of cost, time and ease of catalyst synthesis.

**Table 2:** Probing the robustness of M(OBn)_2_ salts in *rac*-LA ROP at high monomer loadings^a^

Entry	Cat.	LA equiv.	Time (min)	Conv.^b^ (%)	*k*_obs_ (min^−1^)	*M*_n calc_ c (kg mol^−1^)	*M*_n obs_ d (kg mol^−1^)	*M*_n obs_/*M*_n calc_ (2 chains)	*Đ*
1	Zn(OBn)_2_	300	0.5	73	2.53	15.8	15.2	0.96	1.19
2	Zn(OBn)_2_	600	0.5	50	1.29	21.6	20.7	0.96	1.18
3	Zn(OBn)_2_	1000	0.5	44	0.90	31.8	19.0	0.60	1.22
4^e^	Zn(OBn)_2_	1000	0.5	29	0.94	20.6	20.8	1.01	1.14
5^e^	Zn(OBn)_2_	1000	3.5	96	0.94	68.9	49.3	0.72	1.33
6^e^	Zn(OBn)_2_	2500	1	37	0.23	66.1	58.0^f^	0.88	1.14
7^e^	Zn(OBn)_2_	2500	10	90	0.23	161.4	157.5^f^	0.98	1.23
8	Mg(OBn)_2_	500	5	46	0.14	16.6	12.4	0.75	1.37
9	Mg(OBn)_2_	1670	5	11	0.03	13.2	—	—	—
10	Mg(OBn)_2_	1670	80	88	0.03	105.8	25.0	0.24	1.70
11	Ca(OBn)_2_	300	1	4	—	0.85	—	—	—
12	Ca(OBn)_2_	300	25	17	—	3.6	—	—	—

a *rac*-LA pre-stirred in toluene for 30 min prior to addition of M(OBn)_2_, [*rac*-LA] = 1 M in toluene, 70 °C.

b Conversion calculated using ^1^H NMR spectroscopy.

c *M*_n calc_ of polymers calculated from *M*_n calc_ = *M*_0_ × ([*M*]/[*I*]) × conversion assuming 2 chains per catalyst.

d *M*_n obs_ and *Đ* determined by SEC using polystyrene standards in THF. Values corrected by a correction factor of 0.58.^21^

e [*rac*-LA] = 2 M in toluene.

f Uncorrected *M*_n_ reported as correction factor only valid at lower *M*_n_ (<20 kg mol^−1^).^22^

**Fig. 2:**
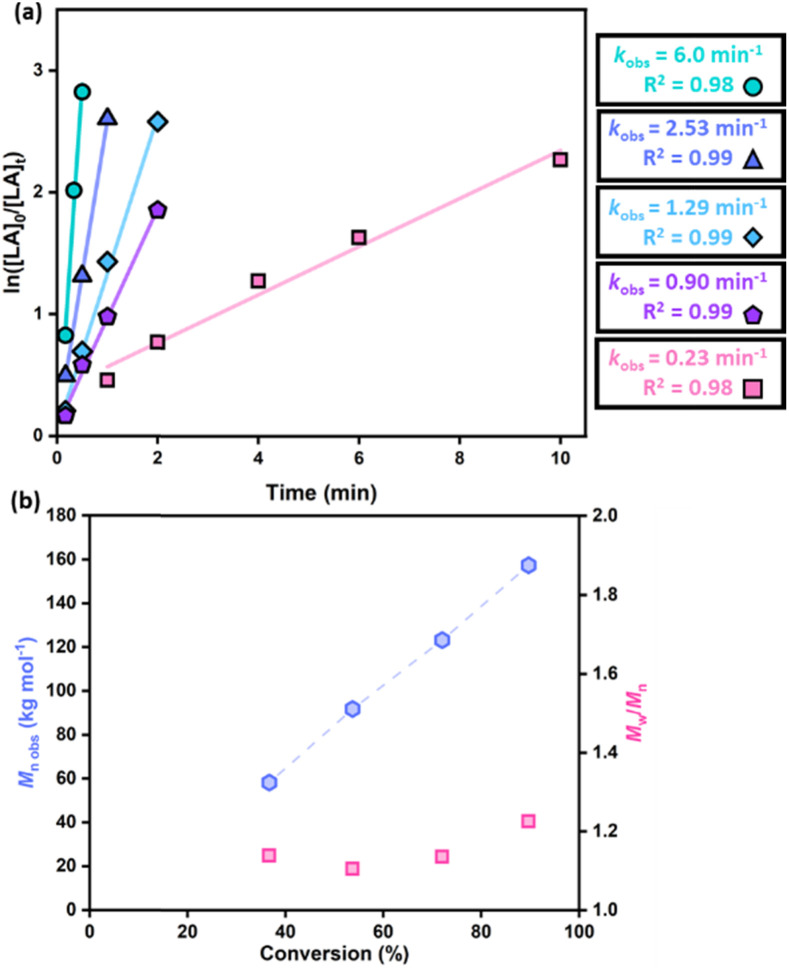
(a) Semi-logarithmic plot of *rac*-LA conversion *vs.* time at 70 °C with Zn(OBn)_2_ in toluene solvent with various [cat] : [*rac*-LA] loading ratios ([*rac*-LA] = 1 M for 1 : 100 (circles); 1 : 300 (triangles); 1 : 500 (diamonds) and 1 : 1000 (pentagon) loadings, and 2 M for 1 : 2500 (square) loadings), with *k*_obs_ and *R*^2^ values presented in the figure legend. (b) Evolution in *M*_n_ and *M*_w_/*M*_n_ with monomer conversion for a [Zn(OBn)_2_] : [*rac*-LA] ratio of 1 : 2500 ([*rac*-LA] = 2 M) (refer to ESI, Fig. S4,† for additional plots of *M*_n_*vs. M*_w_/*M*_n_).

The high activities of these M(OBn)_2_ salts, upon the incorporation of a pre-stir, also makes them highly competitive compared to other reported alkoxide salts. For example, reported Ge alkoxides were found to have extremely low activities, requiring 1 week to reach 90% conversion of LA under harsh reaction conditions (120 °C in chlorobenzene).^38^*In situ* generated Ca(OR)_2_ (R = ^i^Pr, Me) and Y(OR)_3_ (R = ^i^Pr) exhibited high activities, where Ca(OR)_2_, formed from Ca[N(SiMe_3_)_2_]_2_(THF)_2_ and 2-propanol converted 100 equiv. LA in 30 min (r.t., THF, *Đ* = 1.05).^21,25^ Additionally, Y(OR)_3_ formed *via* the exchange of bulky phenolate ligands in yttrium tris(2,6-di-*tert*-butylphenolate) with 2-propanol, converted 150 equiv. l-LA in 8 min (r.t. in dichloromethane, *Đ* = 1.37). By comparison, Zn(OBn)_2_ performs well converting 925 equiv. of LA after just 1 minute (70 °C in toluene, Table 2 entry 6).

Compared to Zn(OBn)_2_, Mg(OBn)_2_ and Ca(OBn)_2_ were less robust at higher monomer loadings. While Mg(OBn)_2_ retained good activities up to a monomer loading of 1670 equiv. *rac*-LA, converting 1470 equiv. in 80 min (Table 2, entries 8–10, Fig. S10†), the *M*_n obs_ values were significantly lower than the *M*_n calc_ values and around 20% of the targeted *M*_n_ values. This may arise from protic impurities, which are more prevalent at higher monomer loadings and can act as chain transfer agents or cause early termination.^36^ For Ca(OBn)_2_, the catalyst activity was rapidly reduced, with a higher [cat] : [*rac*-LA] loading of 1 : 300 giving only 17% conversion after 25 min, compared to 87% in 2 min for a 1 : 100 loading (Table 2, entry 12 *vs.*Table 1 entry 8). The sensitivity of group 2 metal alkoxides, especially the heavier analogues, towards higher monomer loadings has been highlighted in literature. While commercially available Sr(O^i^Pr)_2_ converted 20 equiv. of LA in 1 min at a 1 : 20 [cat] : [l-LA] loading (r.t., toluene),^39^ the activity was significantly lower at higher loadings of 1 : 100, or 1 : 200, converting ∼100 equiv. in 15 min or 90 min, respectively. Unlike the alkaline earth metal analogues, Zn(OBn)_2_ shows promise for maintaining high activities and good polymerisation control at high monomer loadings. While none of the M(OBn)_2_ salts displayed any stereocontrol, producing only atactic PLA from *rac*-LA (*P*_i_ ≈ 0.5), Zn(OBn)_2_ generated isotactic PLA from l-LA (*P*_i_ = >0.99, Table 3, entry 1). This lack of epimerisation is industrially relevant because isotactic PLA delivers superior mechanical properties compared to atactic PLA.

**Table 3:** Employing Zn(OBn)_2_ in LA ROP under industrially relevant conditions

Entry	LA equiv.	Time (min)	Conv.^a^ (%)	*M*_n calc_ b (kg mol^−1^)	*M*_n obs_ c (kg mol^−1^)	*M*_w_ (kg mol^−1^)	*Đ*
1^d^	100	0.5	95	6.8	9.8	—	1.34
2^e^	100	0.5	96	6.9	4.5	—	1.46
3^f^	100	3	75	5.4	6.3	—	1.20
4^g^	2500	30	60	108.0	88.0^h^	154.2	1.75
5^g^	2500	60	72	129.6	90.2^h^	167.0	1.85
6^g^	2500	90	75	135.0	78.6^h^	147.0	1.87

a Conversion calculated using ^1^H NMR spectroscopy.

b *M*_n calc_ of polymers calculated from *M*_n calc_ = *M*_0_ × ([*M*]/[*I*]) × conversion assuming 2 chains per catalyst.

c *M*_n obs_, *M*_w_ and *Đ* determined by SEC using polystyrene standards in THF with a correction factor of 0.58.^22^

d [l-LA] = 1 M in toluene, 70 °C.

e [*rac*-LA] = 1 M in toluene, 70 °C, non-anhydrous conditions.

f [*rac*-LA] = 1 M in toluene, 70 °C, non-anhydrous conditions, technical grade *rac*-LA.

g 150 °C, neat, recrystallised *rac*-LA.

h Uncorrected values reported as correction factor only valid at lower *M*_n_ (<20 kg mol^−1^).^22^

Further demonstrating the industrial relevance of Zn(OBn)_2_, excellent rates for *rac*-LA ROP were maintained when performing the polymerisations in air (see ESI† for details). In 30 s, 96 equiv. of *rac*-LA were converted to PLA, which was identical to the polymerisations performed under an inert atmosphere ([cat] : [*rac*-LA] = 1 : 100, toluene, 70 °C; Table 3, entry 2 *vs.*Table 1, entry 5). In LA ROP, it is common academic practise to purify the monomer *via* multiple recrystallisations followed by sublimation. Here, Zn(OBn)_2_ tolerated unpurified, technical grade *rac*-LA (96% purity), giving a high conversion of 75% in 3 min under air ([cat] : [*rac*-LA] = 1 : 100, toluene, 70 °C; Table 3, entry 3 and Fig. S12†). The high catalyst activities, similar *M*_n_ values and moderate dispersities suggest that Zn(OBn)_2_ is relatively robust towards oxygen, water and other impurities (Table 3, entry 2 and 3). A particular challenge in LA ROP is to achieve high *M*_n_ and well controlled polymerisation under industrial ‘bulk’ conditions, *i.e.* in the absence of solvent. The current industrial standard, Sn(Oct)_2_, polymerises LA in a few hours at 180 °C under bulk conditions.^36^ Some simple iron salts have been reported, including halides, acetates, or alkoxides, yet these require unfavourably low monomer to initiator ratios (0.12–1.20 wt% catalyst), high temperatures (∼180 °C) and long reaction times (<48 h) to achieve high monomer conversions.^40,41^ Interestingly, studies with Zn(OBn)_2_ under bulk polymerisation conditions showed high activities, appreciable *M*_n_ values and moderate dispersities, indicating the potential of Zn(OBn)_2_ to be utilised in industrially relevant settings (entries 4–7). For instance, Zn(OBn)_2_ gave 60% conversion of 2500 equiv. of *rac*-LA in 30 minutes, even when the LA was purified solely through recrystallisation (and not *via* sublimation), to reduce and simplify purification procedures. This generated PLA with high *M*_n_ values (88.0 kg mol^−1^) and reasonable dispersity (*Đ* = 1.75, 150 °C, Table 3, entry 4). Notably, PLA with an industrially relevant *M*_w_ value of 167 kg mol^−1^ was produced (Table 3, entry 5).

These studies describe the use of simple Mg(OBn)_2_, Ca(OBn)_2_ and Zn(OBn)_2_ salts as highly active catalysts for LA ROP following the incorporation of a “pre-stir” step into the reaction set-up. While simple metal alkoxide salts often suffer from low polymerisation rates,^42^ here we show that this can be overcome by incorporating a pre-stir step to improve the solubility. In all cases, the M(OBn)_2_ catalysts reached high conversions at a 1 : 100 catalyst : monomer loading (>90%) in less than 5 minutes, and this took less than 30 seconds for the most active catalyst, Zn(OBn)_2_. Furthermore, Zn(OBn)_2_ retained catalytic activity at high monomer loadings (up to 2500 equiv.), delivering industrially relevant PLA with high molecular weight (*M*_n_ > 150 kg mol^−1^) and good polymerisation control (*Đ* = 1.14–1.23). The robustness of Zn(OBn)_2_ allowed technical grade *rac*-lactide to be polymerised in air, thus offering ample potential for application on larger scale in industrially relevant settings.

## Data availability

The data supporting this article have been included as part of the ESI.†

## Conflicts of interest

There are no conflicts to declare.

## Supplementary Material

RA-014-D4RA07747D-s001
