# Supplementary material for: Simple divalent metal salts as robust and efficient initiators for the ring-opening polymerisation of rac-lactide
Source: RSC Adv. 2024 Nov 29;14(51):38079–84. doi: 10.1039/d4ra07747d (PMC11605502; doi:10.1039/d4ra07747d)
Supplement: RA-014-D4RA07747D-s001 [file RA-014-D4RA07747D-s001.pdf]

Supplementary Information (SI) for RSC Advances.
This journal is © The Royal Society of Chemistry 2024

Supporting Information

Simple Divalent Metal Salts as Robust and Efficient Initiators for the Ring-Opening Polymerisation of *rac*-Lactide

Phoebe A. Lowy[a] and Jennifer A. Garden*[a]

[a]EaStCHEM School of Chemistry, The University of Edinburgh, UK

Email address: j.garden@ed.ac.uk

Table of Contents

General Experimental Details 3

Synthesis of **$Mg(OBn)_2$**: 4

Synthesis of **$Ca(OBn)_2$**: 4

Synthesis of **$Zn(OBn)_2$**: 4

Typical Polymerisation Procedures 5

General Experimental Procedure for the ROP of *rac*-LA with **$M(OBn)_2$** and no Pre-Stir 5

General Experimental Procedure for the ROP of *rac*-LA with **$M(OBn)_2$** 5

General Experimental Procedure for the ROP of *rac*-LA with **$Zn(OBn)_2$** in Air 5

General Experimental Procedure for the ROP of *rac*-LA with **$Zn(OBn)_2$** under Bulk Conditions 5

MALDI-ToF Experimental Data 6

Polymerisation Data 8

References 14

General Experimental Details

All manipulations involving air- or moisture-sensitive compounds were performed either in a glovebox or using standard Schlenk techniques under an argon atmosphere. All reagents and solvents were obtained from Merck, Fisher Scientific, Honeywell and Fluorochem Ltd. and used without further purification unless stated otherwise. Dry solvents (THF, toluene) were collected from a solvent purification system (Innovative Technologies), dried over activated 4 Å molecular sieves and stored under argon. $CDCl_3$, THF-d_8 and toluene-d_8 solvents for NMR spectroscopy studies were degassed by three freeze−pump−thaw cycles and stored over activated 4 Å molecular sieves under argon atmosphere. *Rac*-lactide (*rac*-LA) was purified by double recrystallisation from hot toluene followed by sublimation. Reactions to probe the catalyst efficiency under industrial conditions were performed using *rac*-LA (Sigma-Aldrich, >96% purity by GC) that was used without further purification (referred to as 'technical grade *rac*-LA') or after recrystallisation from hot toluene ('recrystallised *rac*-LA') that was stored at – 20 °C in a glovebox under an argon atmosphere. Benzyl alcohol (BnOH) was dried over CaH_2 and distilled under vacuum prior to use.

^{1}H and ^{13}C spectra were recorded on Bruker AVA500, PRO500 and AVA400 spectrometers at 298 K (500 MHz for ^{1}H and 126 MHz for ^{13}C). Chemical shifts were noted on the δ scale in parts per million (ppm). ^{1}H NMR spectra were measured in a variety of deuterated solvents such as chloroform-*d* ($CDCl_3$) using the residual signal 7.26 ppm and THF-d_8 using the residual signal 1.72 ppm. ^{13}C NMR spectra were measured in a variety of deuterated solvents such as chloroform-*d* ($CDCl_3$) using the residual signal at 77.2 ppm and THF-d_8 using the residual signal at 25.31 ppm. The residual signal of each solvent was used as a reference. For SEC analyses, polymer samples (2 – 10 mg) were dissolved in GPC grade THF (1 ml) and filtered using a 0.2 µm PTFE syringe filter. SEC analyses of the filtered polymer samples were carried out in SEC grade THF at a flow rate of 1 ml min^{-1} at 35 °C on a 1260 Infinity II GPC/SEC single detection system with mixed bed C PLgel columns (300 x 7.5 mm). The RI detector was calibrated using narrow molecular weight polystyrene standards. Mass analysis for the PLA polymers and the three **$M(OBn)_2$** complexes, where M = Mg, Ca and Zn, was carried out using MALDI mass spectrometry. MALDI-ToF MS analyses were performed using a Bruker Daltonics UltrafleXtreme™ MALDI-ToF/ToF MS instrument. The PLA samples to be analysed, dithranol matrix and KI (cationising agent) were dissolved in THF at 10 mg ml^{-1} and the solutions were mixed in a 2:2:1 volume ratio, respectively. A droplet (2 µl) of the resultant mixture was spotted on to the sample plate and submitted for MALDI-ToF MS analysis.

Synthesis of $Mg(OBn)_2$:

$Mg(OBn)_2$ was prepared following a modified literature procedure.[1] BnOH (20.6 µl, 0.20 mmol) was dissolved in dry THF (2.5 ml) in a Schlenk flask. $Mg(HMDS)_2$ (34.2 mg, 0.10 mmol) was subsequently added into the BnOH solution. The resulting mixture was stirred for 16 h at ambient temperature under an argon atmosphere in the glovebox. THF was subsequently removed *in vacuo* resulting in a white powder, which was used directly in polymerisation studies. ^{1}H and ^{13}C NMR spectra of the product were consistent with reported literature values.[1]

^{1}H NMR (500 MHz, THF-d_8): δ 6.99 (br. m 10 H), 4.35 (br. m, 4 H).

^{13}C NMR (126 MHz, THF-d_8): δ 128.40, 126.74, 126.47.

(MALDI-ToF): *m/z*: $[Mg(OBn)]^+$: calculated 131.45; **found 131.915**

Synthesis of $Ca(OBn)_2$:

The synthesis of **$Ca(OBn)_2$** was performed following a literature procedure.[1] BnOH (20.6 µl, 0.20 mmol) was dissolved in THF (2.5 ml) in a Schlenk flask. $Ca(HMDS)_2(THF)_2$ (50.0 mg, 0.10 mmol) was slowly added into the BnOH solution. The resulting mixture was stirred for 16 h at ambient temperature under an argon atmosphere in the glovebox. THF and HMDSH were subsequently removed *in vacuo* resulting in an off-white solid, which was used directly in the preparation of a stock solution for polymerisation studies. ^{1}H and ^{13}C NMR spectra of the product were consistent with reported literature values.[1]

^{1}H NMR (500 MHz, THF-d_8): δ 7.35 (br. d, 4 H), 7.14 (br. t, 4 H), 7.04 (br. t, 2 H), 4.92 (br. d, 2 H), 4.85 (br. d, 2 H).

^{13}C NMR (126 MHz, THF-d_8): δ 128.32, 126.91, 126.41.

(MALDI-ToF): *m/z* $[Ca(OBn)]^+$: calculated 147.22; **found 147.032**

Synthesis of $Zn(OBn)_2$:

$Zn(OBn)_2$ was synthesised following a literature procedure.[1] BnOH (82.4 µl, 0.79 mmol) was dissolved in dry THF (3 ml) in a Schlenk flask. $ZnEt_2$ (48.9 mg, 0.40 mmol) was slowly added into the BnOH solution. The resulting mixture was stirred for 16 h at ambient temperature under an argon atmosphere in the glovebox with the Schlenk tap open to allow ethane gas evolution. THF was subsequently removed *in vacuo* resulting in a white powder, which was used directly in polymerisation studies. The characterisation of **$Zn(OBn)_2$** by NMR spectroscopy was hindered by the low solubility of **$Zn(OBn)_2$** in common NMR solvents, as has been previously reported.[1]

(MALDI-ToF): *m/z* $[Zn(OBn)_2 + H]^+$: calculated 279.67; **found 279.196**

Typical Polymerisation Procedures

General Experimental Procedure for the ROP of *rac*-LA with $M(OBn)_2$ and no Pre-Stir

In a glove box, in an air-tight vial with a magnetic stirrer bar, *rac*-LA (2 mmol) and **$M(OBn)_2$** (0.02 mmol, M = Mg, Ca or Zn) were dissolved in dry toluene (2 ml). The reaction was subsequently stirred using DrySyn heating blocks at the appropriate temperature for the required time. Upon completion, the reaction was quenched in excess chloroform. The volatiles were removed under compressed air and an aliquot was dissolved in $CDCl_3$ for NMR spectroscopic analysis. Reactions were performed in duplicate to ensure data reproducibility.

General Experimental Procedure for the ROP of *rac*-LA with $M(OBn)_2$

In a glovebox, *rac*-LA (2 mmol) was added to an air-tight vial with a magnetic stirrer bar and suspended in dry toluene (1.5 ml). In a second vial, **$M(OBn)_2$** (0.02 mmol, M = Mg, Ca or Zn) was dissolved in dry toluene (0.5 ml each). To ensure *rac*-LA dissolution, both before and during polymerisation, the two solutions were stirred for 30 min at 70 °C using DrySyn heating blocks before the catalyst solution was added to the *rac*-LA solution. The reaction was subsequently stirred for the required time. Upon completion, the reaction was quenched in excess chloroform. The volatiles were subsequently removed under compressed air and an aliquot was dissolved in $CDCl_3$ for NMR spectroscopic analysis. Reactions were performed in duplicate to ensure data reproducibility.

General Experimental Procedure for the ROP of *rac*-LA with $Zn(OBn)_2$ in Air

In a glovebox, *rac*-LA (2 mmol) was added to an air-tight vial with a magnetic stirrer bar and suspended in dry toluene (1.5 ml). In a second vial, **$Zn(OBn)_2$** (0.02 mmol) was dissolved in dry toluene (0.5 ml). Both vials were removed from the glovebox, to ensure *rac*-LA dissolution, and the two solutions were stirred for 30 min at 70 °C using DrySyn heating blocks. Following this, both lids were removed to expose both solutions to air. The catalyst solution was added to the *rac*-LA solution and the reaction was subsequently stirred for the required time. Upon completion, the reaction was quenched in excess chloroform. The volatiles were subsequently removed under compressed air and an aliquot was dissolved in $CDCl_3$ for NMR spectroscopic analysis. Reactions were performed in duplicate to ensure data reproducibility.

General Experimental Procedure for the ROP of *rac*-LA with $Zn(OBn)_2$ under Bulk Conditions

For a polymerisation with a [cat]:[LA] ratio of 1:2500, an air-tight vial with a magnetic stirrer bar was filled with recrystallised *rac*-lactide (50 mmol) and **$Zn(OBn)_2$** (0.02 mmol) in a glovebox. The vial was sealed and placed into a pre-heated DrySyn heating block at 150 °C. The reaction time was started when the reaction mixture was completely molten. A stirring speed of 500 rpm was maintained

throughout the polymerisation. For kinetic measurements, aliquots were withdrawn with a pipette, quenched in excess chloroform and the conversion was determined by ^{1}H NMR spectroscopy in $CDCl_3$.

MALDI-ToF Experimental Data

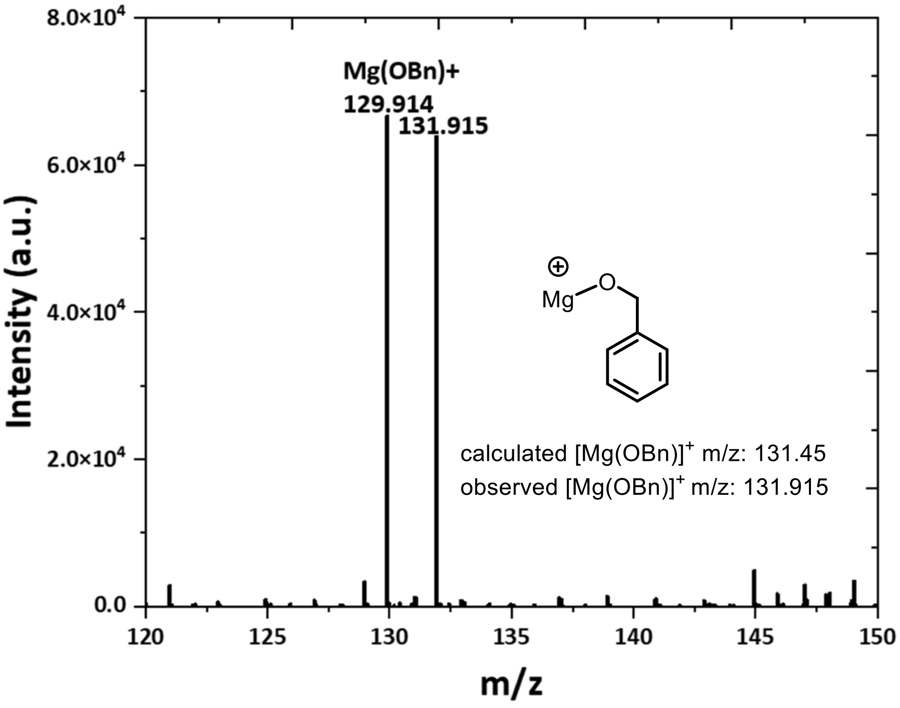

Figure S1: MALDI-ToF spectra of isolated **Mg(OBn)₂**.

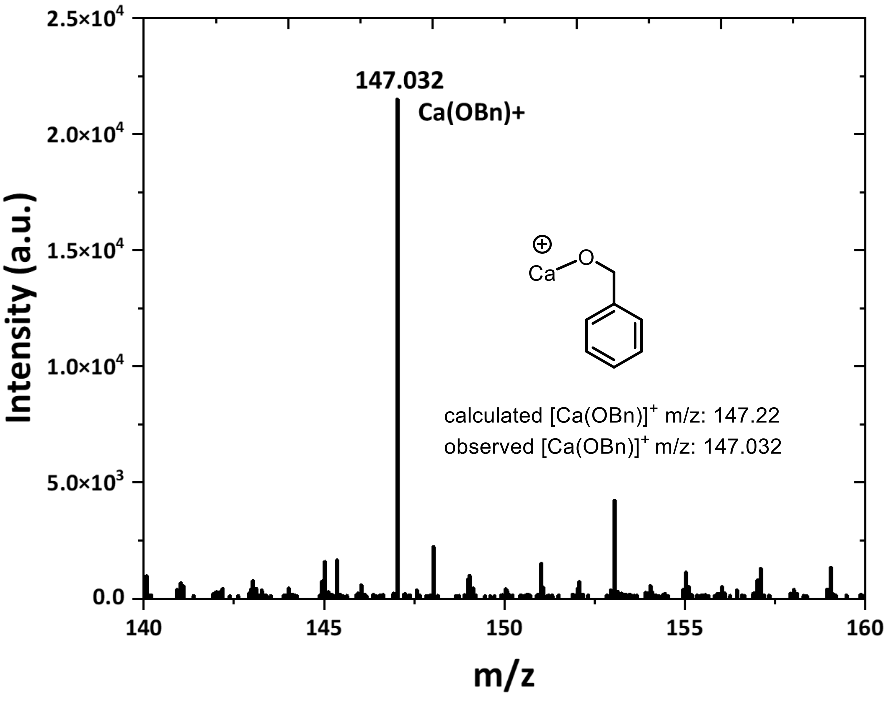

Figure S2: MALDI-ToF spectra of isolated **Ca(OBn)₂**.

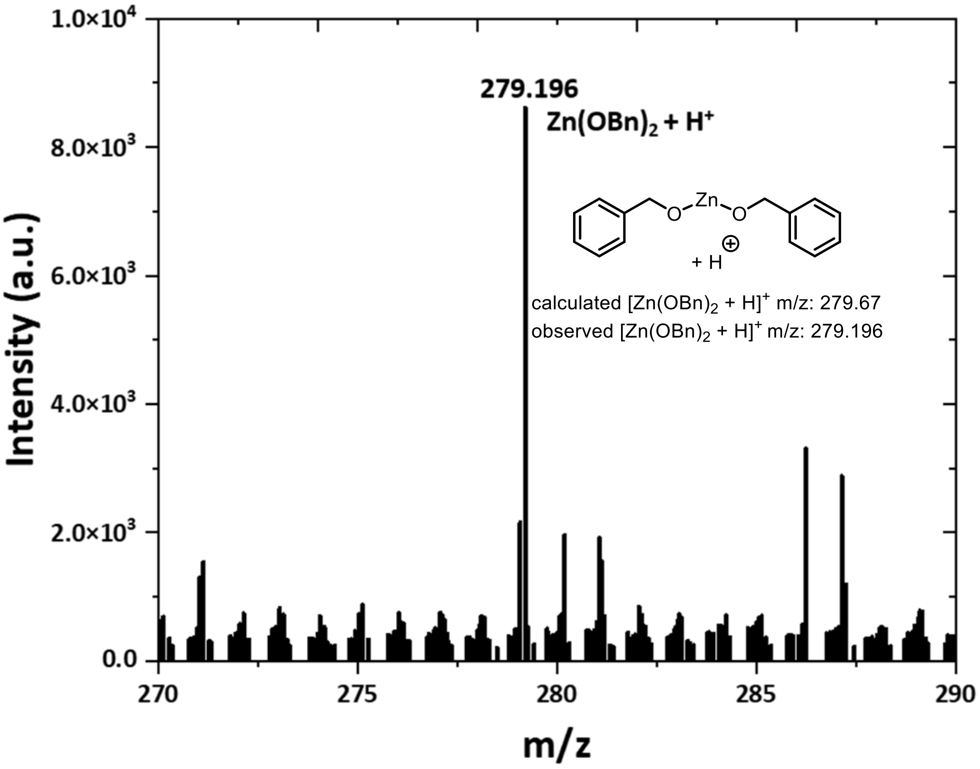

Figure S3: MALDI-ToF spectra of isolated **$Zn(OBn)_2$**.

Polymerisation Data

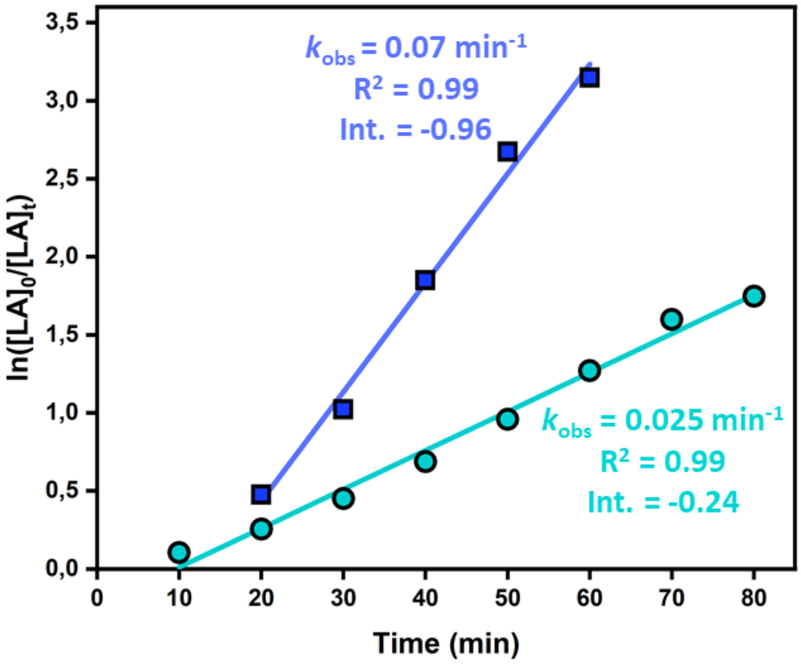

Figure S4: Semi-logarithmic plot of *rac*-LA conversion *vs.* time at 70 °C with **Mg(OBn)$_2$** ■ and **Zn(OBn)$_2$** ● in toluene solvent with a loading ratio [cat]:[*rac*-LA] = 1:100, displaying an induction period of approximately 10 min in the absence of a "pre-stir" step.

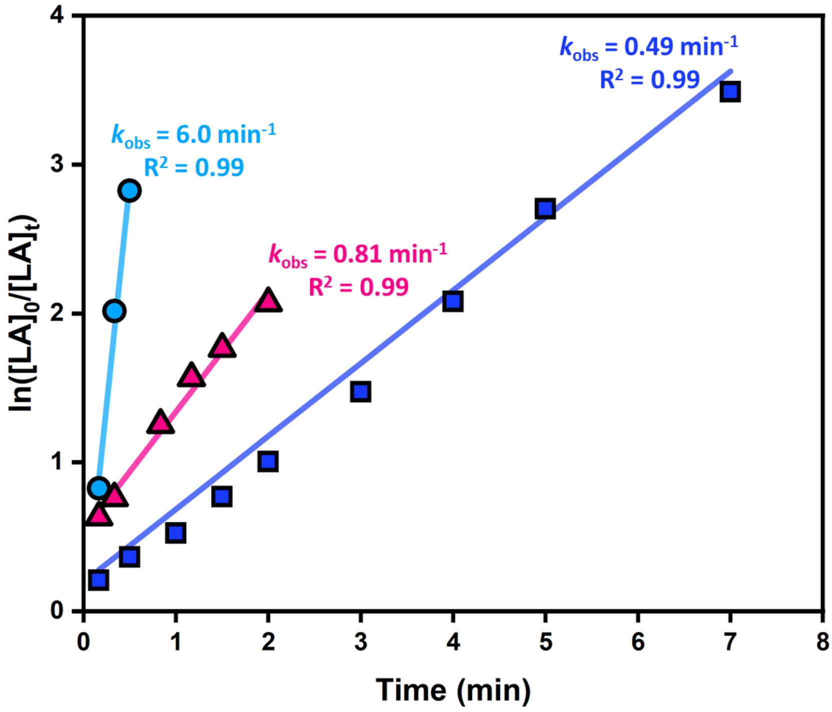

Figure S5: Semi-logarithmic plot of *rac*-LA conversion *vs.* time at 70 °C with **Zn(OBn)$_2$** ●, **Ca(OBn)$_2$** ▲ and **Mg(OBn)$_2$** ■ following a "pre-stir" of *rac*-LA in toluene solvent for 30 min before addition of the initiator, with a loading ratio [cat]:[*rac*-LA] = 1:100 (Table 1, entries 5, 7 and 8).

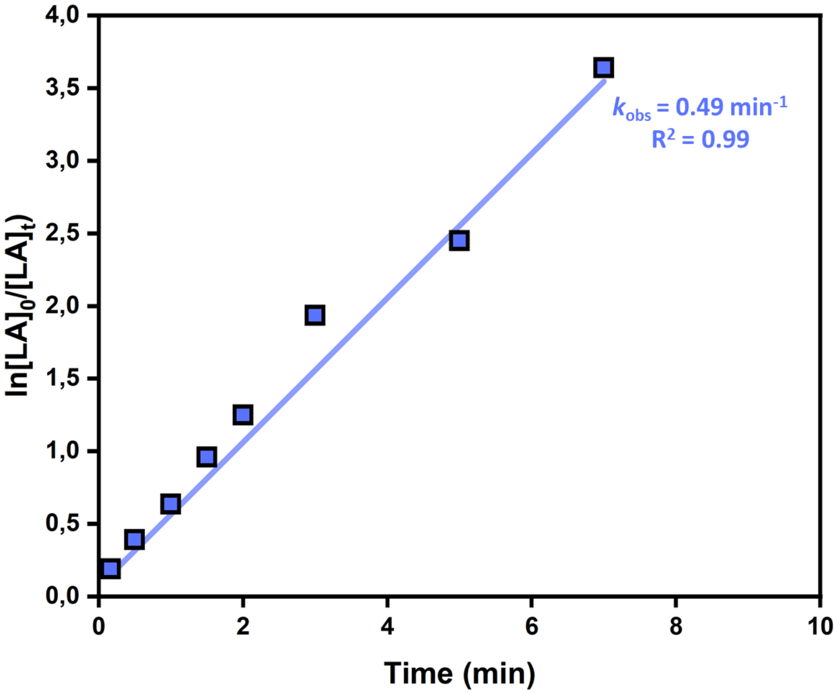

Figure S6: Semi-logarithmic plot of *rac*-LA conversion *vs.* time at 70 °C with **Mg(OBn)₂**, with a 30 min "pre-stir" and a loading ratio of [cat]:[*rac*-LA] = 1:167 (Table 1 , entry 6).

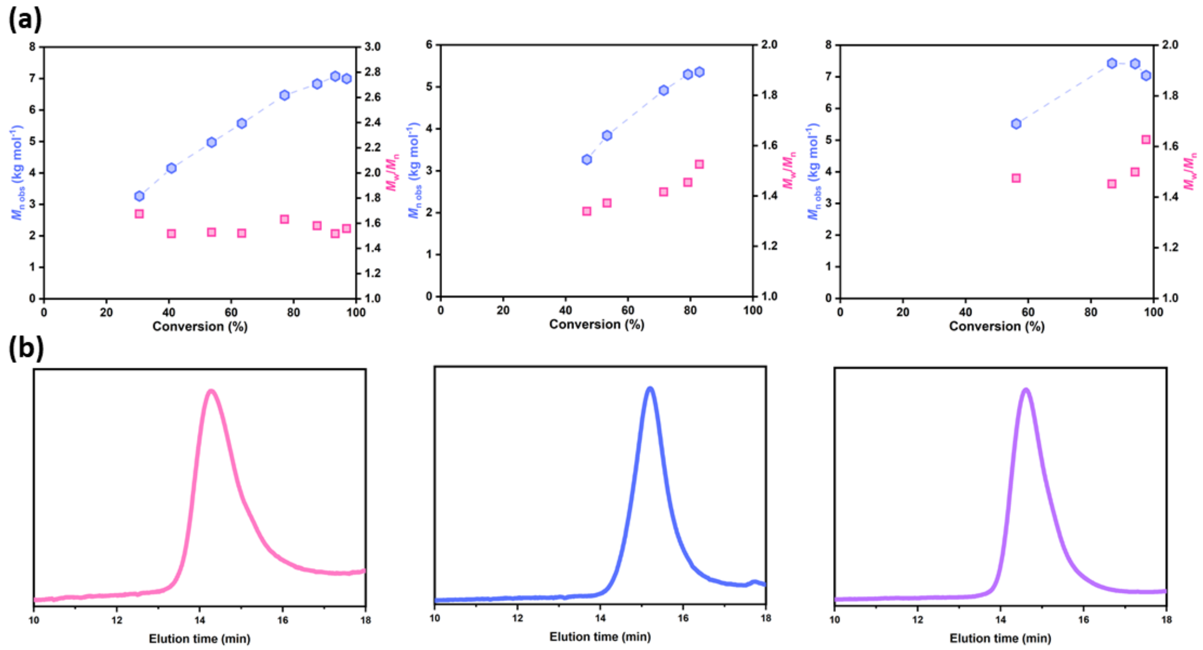

Figure S7: (a) Evolution in M_n and M_w/M_n with monomer conversion of **M(OBn)$_2$** following a "pre-stir" of *rac*-LA in toluene solvent for 30 min before addition of the initiator, with a loading ratio [cat]:[*rac*-LA] = 1:100. From left to right, top row, M = Mg (Table 1, entry 7), Ca (entry 8) or Zn (entry 5), respectively. (b) Example SEC trace of PLA generated with **M(OBn)$_2$** in toluene at 70 °C. From left to right, bottom row, M = Mg (Table 1, entry 6), Ca (entry 8) or Zn (entry 5), respectively).

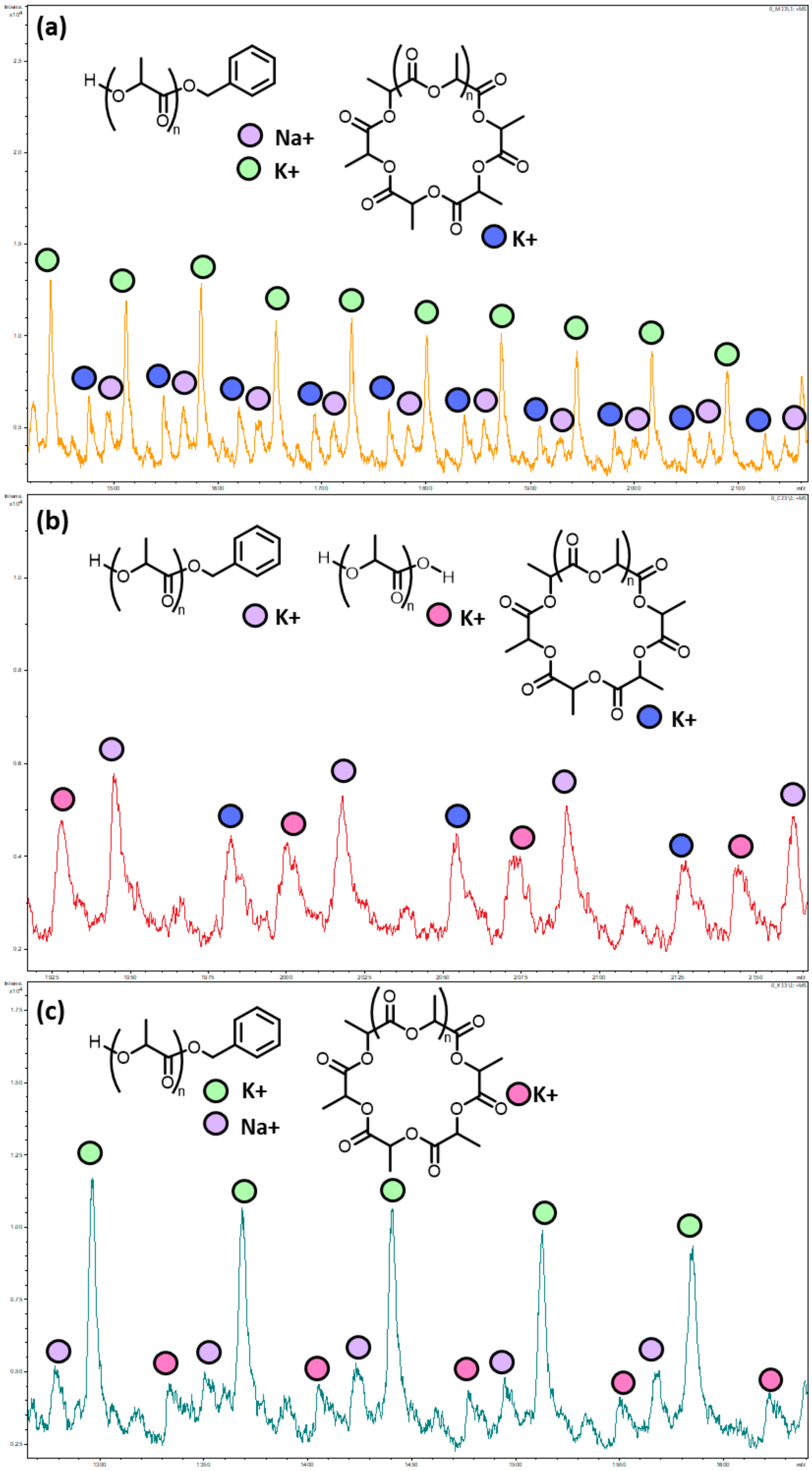

Figure S8: Example MALDI-ToF spectra of PLA resulting from **(a)** 98 % conversion of *rac*-LA in the presence of **Mg(OBn)$_2$** (toluene, 70 °C, top), **(b)** 87 % conversion of *rac*-LA in the presence of **Ca(OBn)$_2$** (toluene, 70 °C) (middle) and **(c)** 98 % conversion of *rac*-LA in the presence of **Zn(OBn)$_2$** (toluene, 70 °C) (bottom).

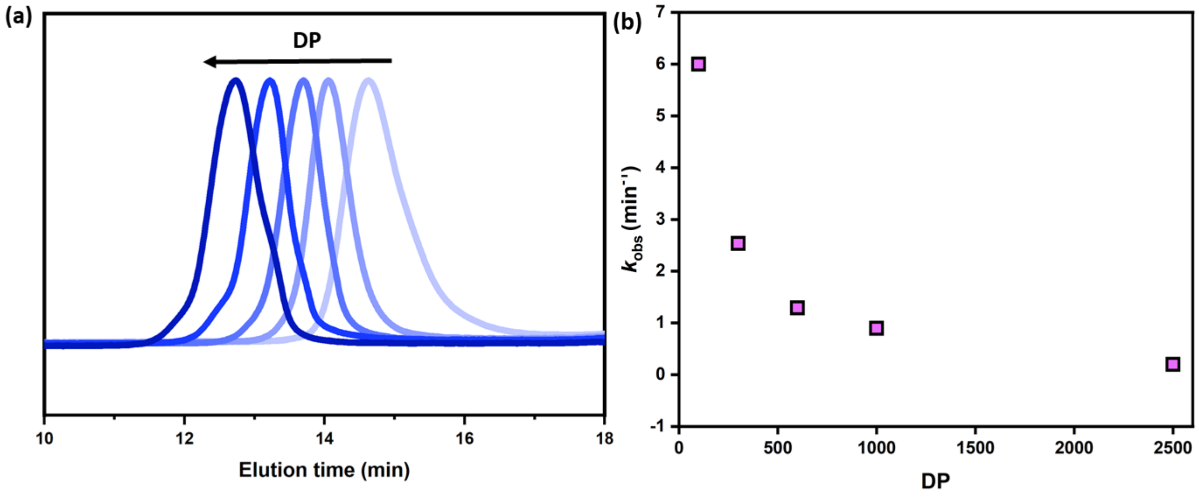

Figure S9: (a) THF SEC traces recorded at different monomer loadings (100-2500 equiv.) for the synthesis of PLA using **Zn(OBn)$_2$**. (b) Plot of the observed rate (k_{obs}) *vs* targeted degree of polymerisation (DP).

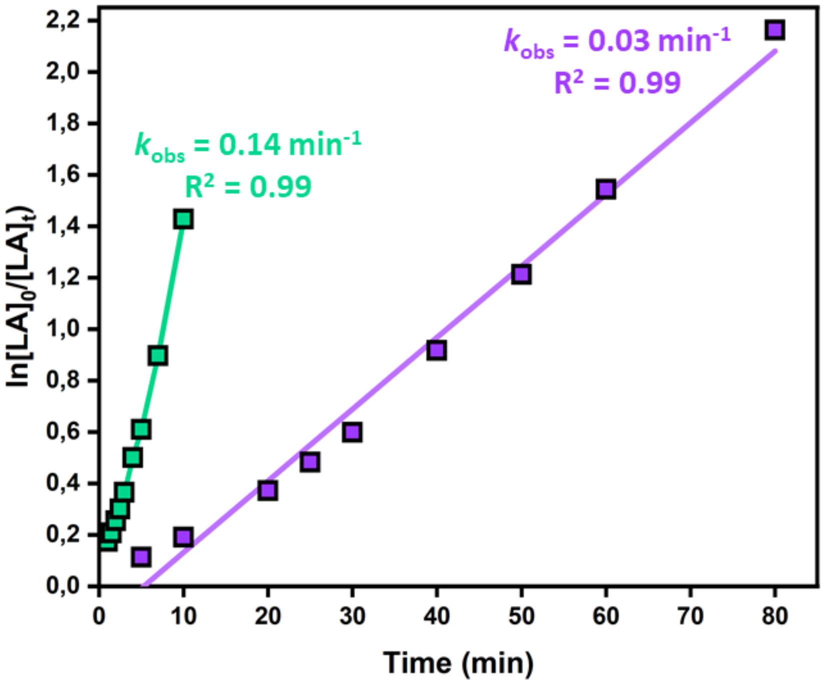

Figure S10: Semi-logarithmic plot of *rac*-LA conversion *vs.* time at 70 °C with **Mg(OBn)$_2$** in toluene solvent with a monomer loading ratio as follows; [cat]:[*rac*-LA] = 1:500 or 1:1670

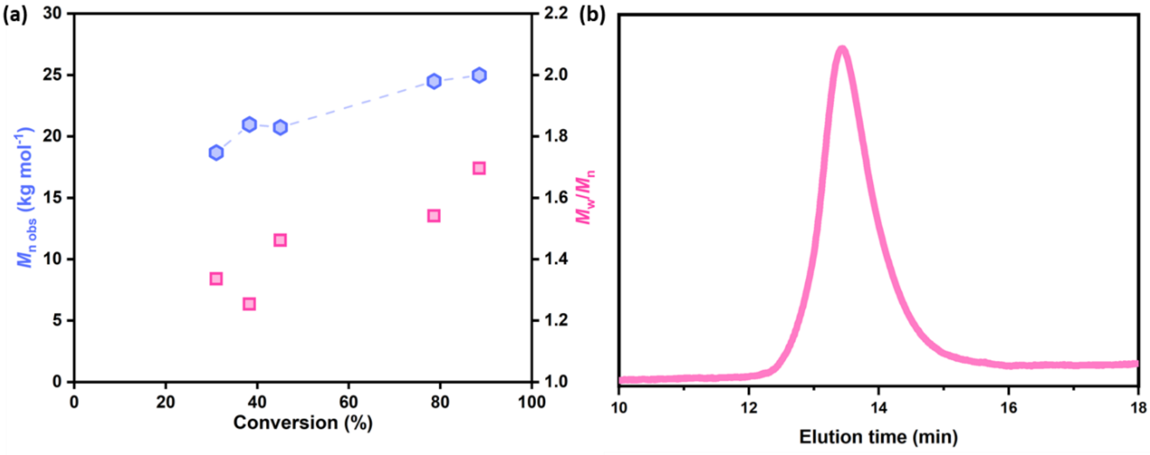

Figure **S11**: (a) Evolution in $\boldsymbol{M_n}$ and $\boldsymbol{M_w/M_n}$ with monomer conversion using **Mg(OBn)$_2$**, with a loading ratio of [cat]:[*rac*-LA] = 1:1670, toluene, 70 ℃, (b) Example SEC trace of PLA obtained from 88 % conversion of *rac*-LA in the presence of **Mg(OBn)$_2$**.

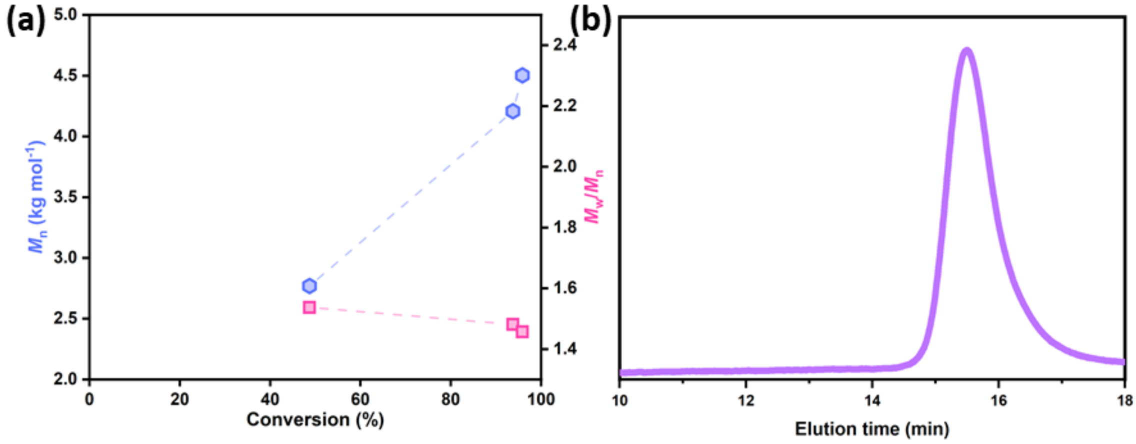

Figure **S12**: (a) Evolution in $\boldsymbol{M_n}$ and $\boldsymbol{M_w/M_n}$ with monomer conversion using **Zn(OBn)$_2$**, with a loading ratio of [cat]:[*rac*-LA] = 1:100, toluene, 70 ℃ in air, (b) Example SEC trace of PLA generated in the presence of **Zn(OBn)$_2$** in air.

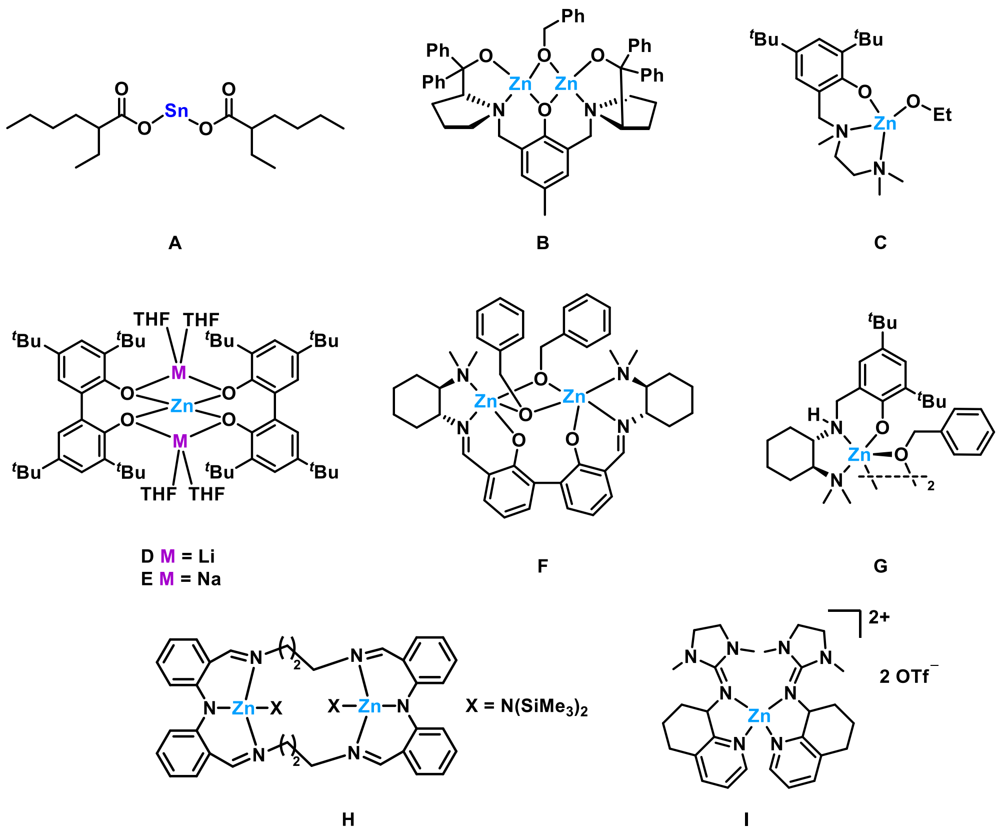

Figure S13: Structures of Sn(Oct)$_2$ **A** and example Zn catalysts **B-I** reported in literature for LA ROP.[2-8]

Table S1: Example Zn-based literature catalysts for the ROP of *rac*-LA and their associated rates. [a]polymerisation performed in air using a non-purified monomer source, [b]non-purified, technical grade LA used.

Catalyst	Solvent	Temperature (°C)	[cat]:[LA]	Time	Conversion (%)	k_{obs} reported	[c]TOF (s^{-1})
**B**[2]	toluene	60	1:100	20 sec	66	3.5 x 10^{-2} s^{-1}	3.30
**C**[3]	DCM	25	1:1000	13 min	96	0.8 x 10^{-3} s^{-1}	1.23
**D**[4]	Toluene	90	1 :175	48 h	87	-	8.81 x 10^{-4}
**E**[a4]	toluene	90	1:175	48 h	94	-	9.52 x 10^{-4}
**F**[5]	DCM	25	1:600	24 h	89	-	6.18 x 10^{-3}
**G**[6]	DCM	25	1:1000	10 min	99	-	1.65
**H**[7]	THF	25	1:1000	30 sec	73	2.82 s^{-1}	24.33
**I**[b8]	-	150	1:1000	166 sec	80	3.8 x 10^{-2} s^{-1}	4.82

References

1. W. Gruszka and J. A. Garden, *Chem. Commun.*, 2022, **58**, 1609–1612.
2. W. Gruszka, L. C. Walker, M. P. Shaver and J. A. Garden, *Macromolecules*, 2020, **53**, 4294–4302.
3. C. K. Williams, L. E. Breyfogle, S. K. Choi, W. Nam, Young Victor G., M. A. Hillmyer and W. B. Tolman, *J. Am. Chem. Soc.*, 2003, **125**, 11350–11359.
4. L. Wang, X. Pan, L. Yao, N. Tang and J. Wu, *EurJIC*, 2011, **5**, 632–636.
5. S. Soobrattee, X. Zhai, K. Nyamayaro, C. Diaz, P. Kelley, T. Ebrahimi and P. Mehrkhodavandi, *Inorg. Chem.*, 2020, **59**, 5546–5557.
6. T. Ebrahimi, E. Mamleeva, I. Yu, S. G. Hatzikiriakos and P. Mehrkhodavandi, *Inorg. Chem.*, 2016, **55**, 9445–9453.
7. A. Thevenon, C. Romain, M. S. Bennington, A. J. P. White, H. J. Davidson, S. Brooker and C. K. Williams, *Angew. Chem. Int. Ed.*, 2016, **55**, 8680–8685.
8. A. Hermann, T. Becker, M. A. Schäfer, A. Hoffmann and S. Herres-Pawlis, *ChemSusChem*, 2022, **15**, e202201075.
